# Optimal Limestone Content on Hydration Properties of Ordinary Portland Cement with 5% Ground Granulated Blast-Furnace Slag

**DOI:** 10.3390/ma17133255

**Published:** 2024-07-02

**Authors:** Ingyu Kang, Sangchul Shin, Jinman Kim

**Affiliations:** 1 Department of Architectural Engineering, Kongju National University, 1223-24 Cheonan-Daero, Cheonan City 330-717, Chungcheongnam-do, Republic of Korea; kik4098@naver.com; 2 Environment-Friendly Concrete Research Institute, Kongju National University, 1223-24 Cheonan-Daero, Cheonan City 330-717, Chungcheongnam-do, Republic of Korea; hiykhj@kongju.ac.kr

**Keywords:** Portland cement, limestone, carbon neutrality, clinker, hydration reaction, supplementary cementitious materials

## Abstract

In this study, the effect of limestone content on the mechanical performance and the heat of hydration of ordinary Portland cement (OPC) was investigated. Changes in the phase assemblage were analyzed through XRD and thermodynamic modeling. The purpose of the study was to identify the optimal limestone content in OPC. As a result of the experiment, all samples were found to have equal fluidity. Increasing the limestone content accelerated the hydration of the cement before approximately 13 h and shortened the setting time due to the acceleration of the initial hydration reaction. The compressive strength of the cement mortar showed a dilution effect, with lower compressive strength compared to the reference sample at an early age, but it gradually recovered at a later age. This is because, as shown in the XRD and thermodynamic modeling results, the carboaluminate phases formed due to the chemical effect of limestone contributed to the development of compressive strength. As a result, within the scope of this study, it is believed that maintaining the limestone content in OPC within 10% is optimal to minimize quality degradation.

## 1. Introduction

The cement industry significantly contributes to global carbon emissions, accounting for approximately 7% of worldwide CO_2_ emissions [1]. A primary source of these emissions is the decarbonation of limestone necessary for clinker production, which constitutes roughly 60% of the total CO_2_ emissions from the cement manufacturing process. Consequently, reducing clinker consumption emerges as an effective strategy for mitigating the overall carbon footprint of cement production. The global cement and concrete industries are actively engaged in developing environmentally friendly cement by decreasing clinker content and using industrial by-products as supplementary cementitious materials (SCMs).

Europe has the widest range of SCMs available for cement manufacturing, and many types of blended cement are already manufactured and used, resulting in a low clinker coefficient compared to other countries [2,3,4,5]. On the other hand, although Korea has established standards for blended cement, its dependence on OPC is still high, at over 80%, compared to Europe, where it is about 30% or less. This suggests that clinker usage and CO_2_ emissions are quite high in Korea. However, in line with the global goal of achieving carbon neutrality by 2050, the Korean cement industry is also trying to reduce its dependence on clinker by increasing the content of SCMs in OPC or through the development of new blended cement. In particular, the overriding goal is to reduce dependence on clinker by increasing the SCM content, which is currently limited to 10% in OPC under the KS standard, to up to 20%. However, this increase in SCM content results in a dilution effect that reduces the reactivity of the cement due to a decrease in clinker, the main reactive component, and must be approached with caution as it is ultimately closely linked to the performance of the concrete structure. Additionally, in the case of Korea, which is highly dependent on OPC, there is a need to intensively explore various SCMs, their optimal ratios, and their impact on reducing carbon emissions while maintaining cement reactivity to solve these problems.

From a materials science perspective, increasing the content of ground granulated blast-furnace slag (GGBFS) as specified in the Korean KS standard presents an optimal strategy to enhance reactivity and mitigate the dilution effect [6]. However, as the steel industry transitions to carbon-neutral production methods, such as electric and hydrogen reduction processes, instead of traditional blast furnace operations, the future availability of GGBFS to support the cement industry’s carbon neutrality goals is expected to be limited. In this context, increasing the limestone content as a substitute for GGBFS is emerging as a feasible alternative that offers both availability and economic advantages [7].

Limestone exhibits lower reactivity compared to other SCMs and does not engage in hydration reactions autonomously. Nevertheless, it participates both directly and indirectly through interactions with C_3_S and C_3_A, the main clinker phases, thereby enhancing the engineering properties of the hardening cement and partially offsetting the dilution effect. The beneficial impact of limestone addition is supported by various theories.

Primarily, the incorporation of limestone in cement initiates the formation of hemicarboaluminate and monocarboaluminate through direct hydration reactions with C_3_A, a mechanism known as the chemical effect [8]. The formation of these CO_3_–AFm phases prevents the transformation of ettringite into monosulfoaluminate post-gypsum depletion, allowing ettringite to persist even in aged cement systems. This contributes to an increase in the volume of hydration products, thereby enhancing porosity and cement strength [9,10].

The limestone not participating in reactions acts as a filler, filling pores and forming diverse microstructures, thereby accelerating initial hydration. These indirect reactions are called the filler effect. In cement, the hydration reaction begins when the cement contacts water; however, limestone does not participate in the hydration reaction before gypsum is consumed. Therefore, a binder suspension with added limestone has a relatively high water-to-clinker ratio, increasing spaces for hydration product precipitation [11]. In these spaces, finely ground limestone acts as nucleation seeds for growing clinker hydrates, accelerating C_3_S hydration after the induction period [12,13]. Consequently, more C–S–H is initially formed, increasing the degree of hydration of C_3_S by enhancing the degree of unsaturation in the pore solution for C_3_S [14].

Many studies have been conducted to investigate the effect of replacing limestone in amounts ranging from as little as approximately 5% to as much as approximately 35% on cement performance. Although there are differences of opinion in the research results, there is a consensus that the engineering properties of cement deteriorate as the limestone content increases, with approximately 15% suggested as the critical point. The performance of cement is determined by the content of added limestone, and since limestone has limited participation in the hydration, it is important to determine the optimal limestone content that minimizes the dilution effect [15,16].

Research on the reaction mechanism of limestone in Portland cement and its replacement for clinker at various levels has been continuously conducted in the past. Alternatively, the goal is to develop Portland limestone cement. Most studies on limestone have focused on blended cement, and there is a lack of data on the effect of increasing limestone content on hydration behavior in OPC. This highlights the need for empirical research to determine the ideal limestone content in OPC.

In particular, increasing the limestone content in OPC is a method that has not been attempted by any country in the world and is only planned for the future by the Portland Cement Association in the United States. While expanding the blended cement market, as seen in Europe, is the most desirable way to promote carbon neutrality in the cement industry, in Korea and the United States, where OPC is the main focus, improving performance by increasing the SCM content in OPC is necessary. There will be a need to promote the use of Portland cement with minimal changes, and this study is expected to make a significant technical contribution in this context.

Therefore, this study aims to analyze the changes in the mechanical performance, the heat of hydration, and the phase assemblage of cement by varying the limestone content in OPC. At this time, 5% GGBFS was used to reduce the dilution effect of cement. The mechanical performance and hydration of cement were investigated using fluidity, setting time, heat of hydration, compressive strength, and drying shrinkage. Changes in the phase assemblage of the hydration product were analyzed through XRD and thermodynamic modeling. Additionally, we aim to identify the optimal limestone content in OPC by comprehensively reviewing the experimental results.

## 2. Materials and Methods

### 2.1. Experimental Design

The OPC distributed in South Korea uses 5% of either GGBFS, fly ash, or pozzolan and contains 10% SCM content, which includes 5% limestone. Therefore, experimental cement was prepared to increase the SCM content by strictly controlling the ratios of the constituent materials. The cement was blended at ratios suitable to the goals of the experiments, as described in Table 1. The term “plain” denotes cement that adheres to the composition ratios specified in the KS L 5201 standard [17]. The labels L7.5 to L15 denote cements where the limestone content was incrementally increased by 2.5% from the plain. The gypsum was set at 5% of the clinker used, and the GGBFS was fixed at 5% in all mixtures. The water-to-binder ratios for both cement paste and mortar were fixed at 50%, with a binder-to-sand ratio of 1:3, as per KS L ISO 679 [18]. Assessments performed on the cement paste included X-ray diffraction and heat of hydration measurements, while the cement mortar was subjected to evaluations of flow, setting time, compressive strength, and drying shrinkage.

### 2.2. Materials

**Figure 1:**
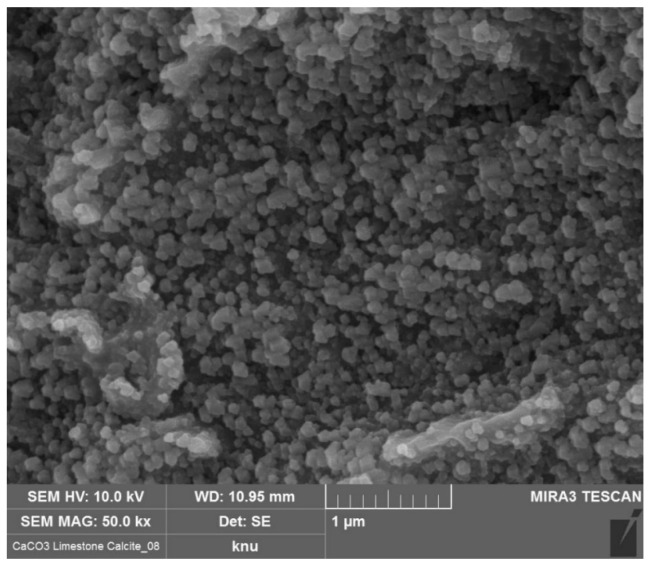
SEM image of limestone.

The clinker used in this study was provided by Sampyo, a cement company in South Korea. For the desulfurized calcium sulfate, which is a by-product of removing sulfur oxides at thermal power plants, this study used gypsum from Hanil, another cement company in South Korea. The slag consisted of GGBFS, made by rapidly cooling high-temperature molten slag with sprayed water, and was received from Hyundai Steel, South Korea. The limestone used was of the same grade as that used as a raw material in clinker manufacturing. The limestone powder’s CaCO_3_ grade was 93%, and it was provided by Halla, a cement company in South Korea. The SEM image of the limestone powder used is shown in Figure 1.

**Figure 2:**
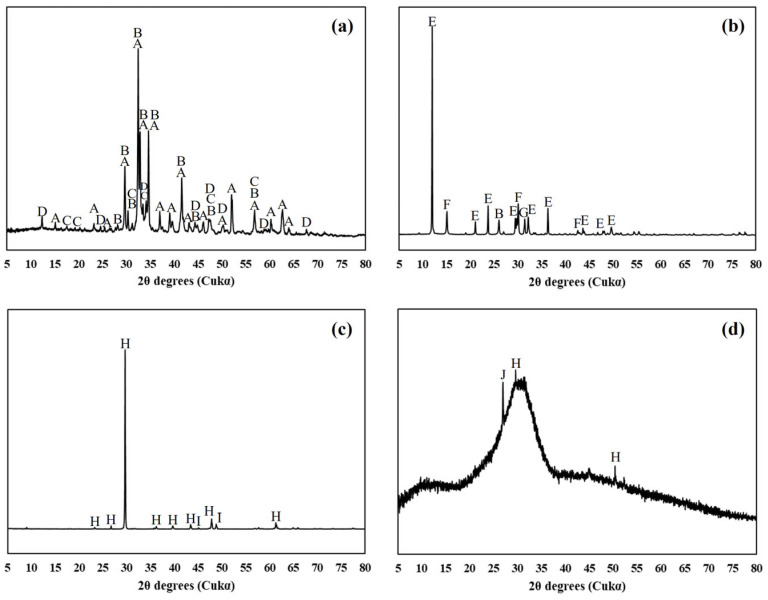
XRD patterns of (**a**) clinker, (**b**) calcium sulfate, (**c**) limestone and (**d**) GGBFS. The peaks of alite (A), belite (B), aluminate (C), ferrite (D), dihydrate (E), hemihydrate (F), anhydrate (G), calcite (H), dolomite (I) and quartz (J) are indicated.

**Figure 3:**
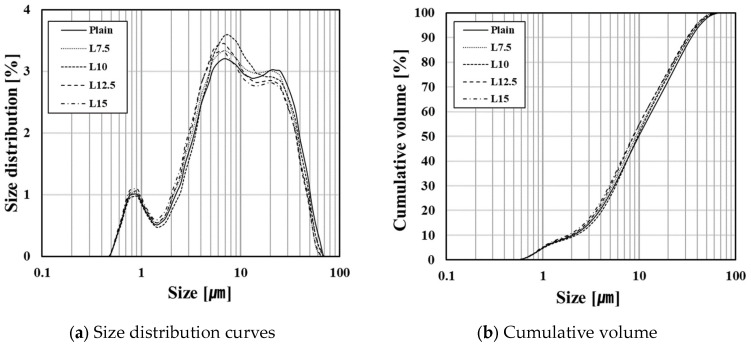
Particle size distribution of manufactured cements.

The chemical compositions of the raw materials were determined via XRF and are shown along with the materials’ densities in Table 2. The XRD patterns of the raw materials used are shown in Figure 2. Table 3 displays the physical properties and chemical compositions of the cement prepared using the raw materials mentioned above. The densities of the prepared cements were 3.14–3.15 g/cm^3^, which are similar to commercially available cement products. Fineness, measured via an air permeability apparatus, was approximately 3600–4000 cm^2^/g and showed a trend of increasing as the limestone content increased. It is believed that the limestone grinding efficiency was higher than that of the clinker because the cement was made via co-grinding, ultimately increasing the fineness of the ground cement. The particle analysis of the prepared cement was performed via laser diffraction in ethanol, considering the reactivity of the cement, and it is shown in Figure 3.

### 2.3. Methods

To prepare the cement, each of the raw materials was crushed to less than 5 mm using a jaw crusher and then dried at 105 ± 5 °C to remove moisture. Subsequently, they were individually weighed according to the ratios set by the experimental design to create a single batch of binder, which was then fed into a ball mill and finely ground. To increase the efficiency of the grinding and prevent the interior of the ball mill from being coated, 0.1% grinding aid (diethylene glycol) was added. For the grinding conditions, the plain was ground for 70 min at 50 rpm to obtain a fineness similar to that of commercially available OPC. Notably, all mixtures were ground under the same conditions. After grinding, the cement was discharged over 5 min at the same rpm, sealed in vinyl to prevent contact with moisture, and then stored.

All mixtures were mechanically blended in constant temperature/humidity conditions at a temperature of 20 ± 3 °C and a relative humidity of 60% in accordance with the KS L ISO 679 standard [18]. Directly after blending, flow was measured by dropping a flow table 25 times within 15 s in accordance with the KS L 5105 standard [19]. To determine the cement setting times, the mortar was shaped with a 100 × 100 × 400 mm prism mold, and the initial and final setting times were set as the times when the penetration resistance was 3.5 MPa and 28 MPa, respectively, in accordance with the KS F 2436 standard [20].

To measure compressive strength, the mortar was placed in a 40 × 40 × 160 mm prism mold, removed after 24 h, and water-cured in a water tank at 20 ± 1 °C. The compressive strength was measured at 1, 3, 7, and 28 days, and the averages of three measurements were used as the test values.

A cement paste with a 0.5 w/b ratio was prepared to perform a qualitative analysis of the hydration of cement according to limestone content. Specimens that were cured for 3, 7, and 28 days were cut into 5 mm sections and soaked in screw-cap vials filled with acetone to halt hydration via a vacuum desiccator. Subsequently, the specimens were dried in a 60 °C dryer to evaporate the acetone, and the dried specimens were finely ground to less than 45 μm. The hydrate analysis was performed via X-ray diffraction using a MiniFlex600 device by Rigaku (Tokyo, Japan) at a measurement range of 5–80° and a scan speed range of 2°/min with a 600 W X-ray generator and the following conditions: CuKα-40 kV-15 mA.

The heat of hydration of cement was measured via isothermal calorimetry using a TAM Air device by TA Instruments. To perform the measurements, 10 g of cement and 5 g of distilled water were placed in a vessel and mixed with a glass rod. In particular, 4 g of paste was collected from this mixture, poured into an ampoule, and then placed in the calorimeter. Distilled water was used as the reference sample in the experiments. Measurements were taken at one-minute increments for 72 h at 21 °C, and the heat flow and total heat were calculated.

The phase assemblages according to the presence and content of limestone were investigated using thermodynamic modeling with the Gibbs free energy minimization method. The thermodynamic data of the hydrates formed by the hydration reaction in the cement system were analyzed through the open-source software GEM-Selektor version 3 using the PSI-GEMS and CEMDATA18 thermodynamic databases [21]. The Debye–Hückel equation for potassium hydroxide was used to calculate the activity of ions in the aqueous solution [22]. The common ion size parameter and the short-range interaction parameter are 3.67 Å and 0.123 kg/mol, respectively. Additionally, modeling for cement hydration was performed at 20 °C and 1 bar. The binders used in the modeling were clinker, gypsum, GGBFS, and limestone, the same materials used in the actual experiments. Clinker was quantified for its mineral composition through XRD-Rietveld refinement for modeling, showing the presence of major mineral phases such as C_3_S, C_2_S, C_3_A, and C_4_AF at 50.1%, 27.6%, 7.1%, and 10.6%, respectively. Gypsum was used at 5% relative to the clinker content, and GGBFS was consistently used at 5% according to KS L 5201 [17]. Limestone was added up to a maximum of 15% to model the phase assemblage according to the chemical effects of limestone. The reactivity of clinker was applied to the model using an empirical approach proposed by Parrot and Killoh [23], which represents the reactivity of clinker minerals as a function of time. The hydration degree of GGBFS was utilized by referencing the reactivity obtained through a simple pozzolanic test conducted according to the method presented by [24]. In [24], the reactivity of GGBFS at 1, 2, 7, 28, and 90 days is presented. Reactivities of 20% and 29% for 7 and 28 days, respectively, were used in the modeling, and for comparison with XRD experimental results, the reactivity at 3 days was estimated to be 12% through log-linear interpolation. All modeling was performed under conditions of w/b 0.5, 20 °C, and 1 bar. The hydrates predicted to form through the hydration reaction were defined as C–S–H, portlandite, ettringite, monosulfoaluminate, hemicarboaluminate, monocarboaluminate, and hydrotalcite. Si-hydrogarnet (Ca_3_(Al_x_Fe_1−x_)_2_(SiO_4_)_y_(OH)_4(3−y)_), which contains aluminum and iron, is a thermodynamically more stable hydrate than monosulfoaluminate and monocarboaluminate under room temperature conditions. However, it is reported to form in Portland cement under high-temperature conditions due to its slow formation rate, and thus it was excluded from this modeling [25,26,27,28].

## 3. Results

### 3.1. Flow

**Figure 4:**
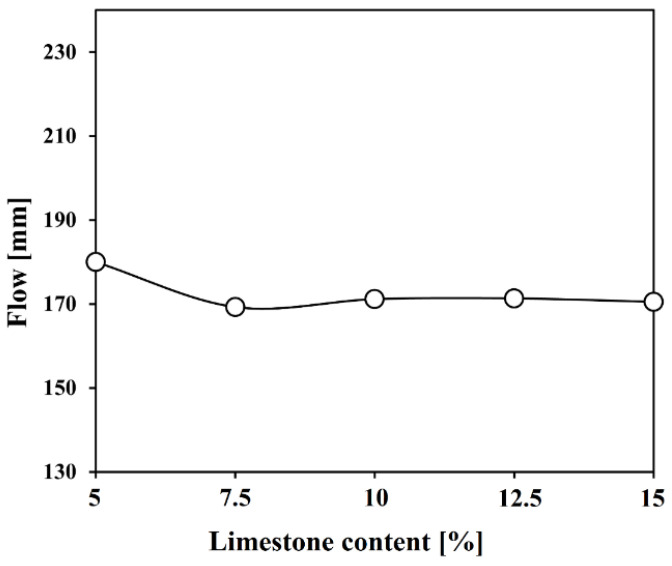
Flow of Lab-OPC with different contents of limestone.

Figure 4 presents the outcomes of flow tests with increasing limestone content. The control mix, designated as “plain”, exhibited the highest flowability with a measurement of 180 mm. For mixes L7.5 to L15, a decrease of approximately 10 mm in flow was noted in comparison to the plain mix. However, these results lack sufficient data to explain the influence of limestone content on fluidity. This is because no change in fluidity was observed even when the limestone content increased from 7.5% to 15%. Additionally, since all samples were co-grinding, it is difficult to separately explain the effect of the particle size of each raw material on fluidity.

### 3.2. Hydration Kinetics

**Figure 5:**
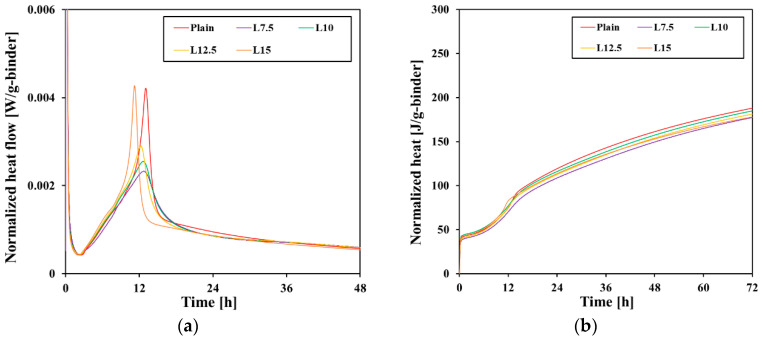
Heat flow (**a**) and total heat (**b**) per gram of binder.

**Figure 6:**
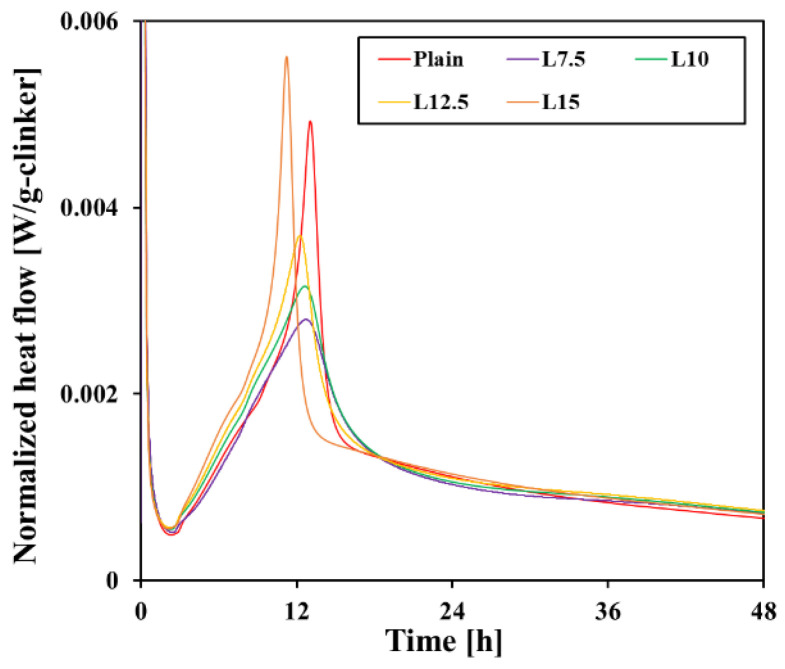
Heat flow per gram of clinker.

Figure 5 shows the heat flow normalized per gram of binder and the total heat per gram of binder according to the increase in limestone content. All samples had an induction period of approximately 3 h, after which the acceleration period began. This is the stage where the hydration of C_3_S among clinker minerals begins in earnest. The heat flow was found to become steeper as the limestone content increased, suggesting that the hydration of C_3_S was promoted. Thereafter, hydration continued, and the time until the main peak of the C_3_S was shortened in all test samples. When the measurement results were calculated as heat flow normalized per gram of clinker, as shown in Figure 6, the C_3_S hydration acceleration effect after the induction period was clearer, and it can be observed that the main peak values also increased [29]. The total heat of the test samples was similar to or higher than that of the plain for approximately 12 h, but then there was a reversal as the total heat of the plain was the highest at 48 h.

The high heat release that occurred before the C_3_S main peak suggests that the initial reaction of C_3_A resulted in the precipitation of initial ettringite. Afterwards, a second peak of C_3_A is generally observed in the form of a shoulder peak after the main peak of C_3_S, at which time additional ettringite is precipitated. However, no distinct C_3_A peak was observed in the experimental results of this study.

### 3.3. Setting Time

**Figure 7:**
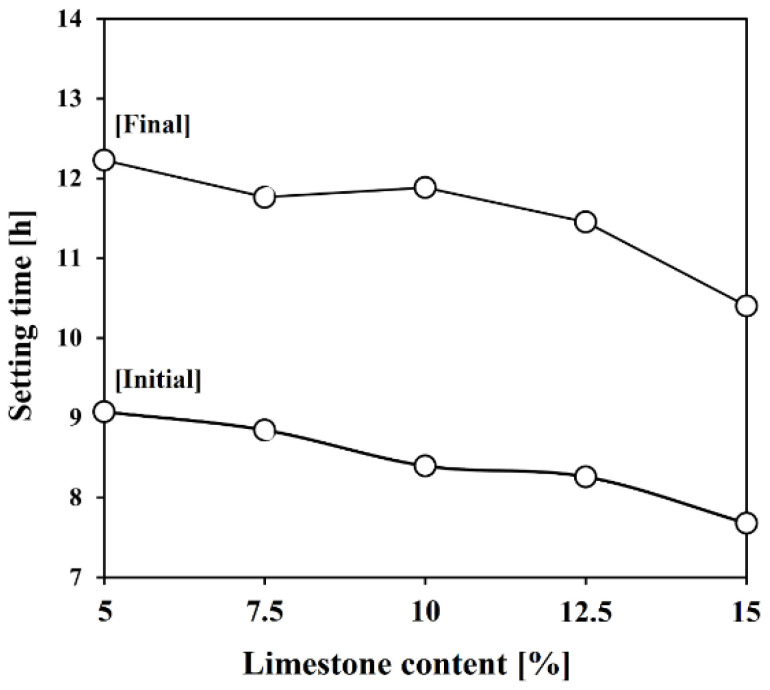
Setting time of Lab-OPC with different contents of limestone.

Figure 7 shows the results of the experiments on setting time according to the increase in limestone content. In the plain mix, the initial setting time occurred at 9 h, and the final setting time occurred at 12.2 h. There was a trend in which the setting time was shortened as the limestone content increased. The fastest setting occurred in L15, where the initial setting time was around 1 h shorter than that of the plain, and the final setting time was approximately 1.8 h shorter.

### 3.4. Compressive Strength Development

**Figure 8:**
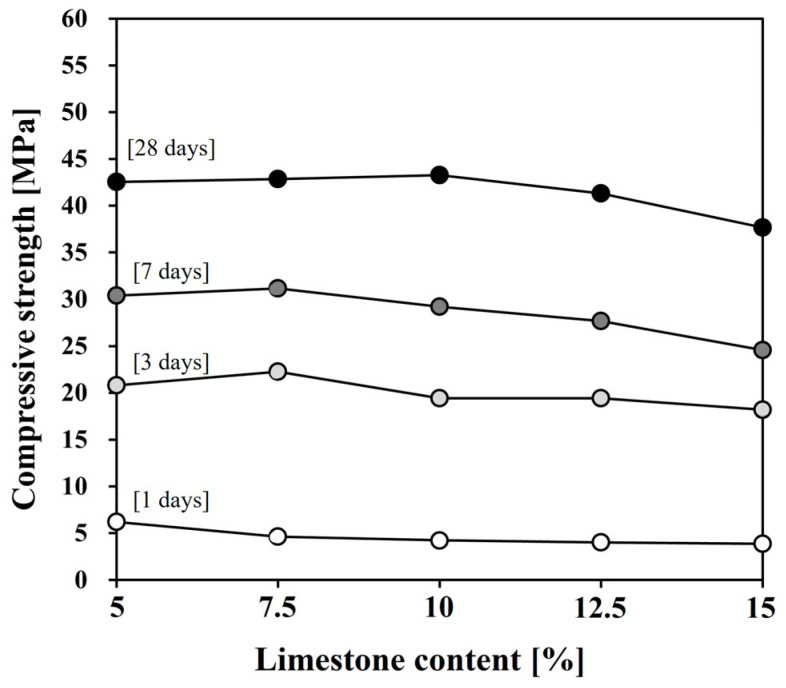
Compressive strength of Lab-OPC with different contents of limestone.

Figure 8 shows the compressive strength measurement results, and Table 4 shows the strength development rate compared to the plain specimen. The test samples’ overall strength properties tended to be similar to or less than those of the plain with increasing limestone content, which is a natural result of the reduction in clinker content, the main reactive material [30]. In particular, at 1 day, all test samples showed 25–40% lower strength development than the plain. However, it can be observed that afterward, the strength development difference in comparison with the plain gradually became smaller as hydration continued. The strength development of L7.5 was higher than that of the plain at all ages after 3 days, and L10 and L12.5 showed strength development of around 90% or more. However, L15 showed a strength development of less than 90% even at 28 days. Therefore, this study showed that the compressive strength did not decrease even when the limestone content was up to 10%.

### 3.5. Phase Assemblages

#### 3.5.1. X-ray Powder Diffraction

**Figure 9:**
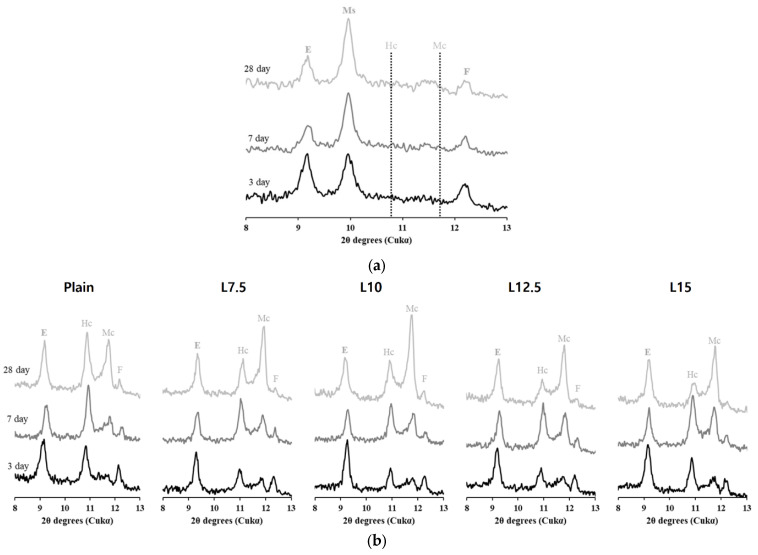
XRD pattern of (**a**) Portland cement (only clinker + gypsum) and (**b**) Lab-OPC (with different contents of limestone) cured for 3, 7, 28 days. Where E: ettringite, Ms: monosulfoaluminate, Hc: hemicarboaluminate, Mc: monocarboaluminate, F: ferrite.

XRD analysis was conducted on a mixture of clinker and gypsum, as well as on five systems used in the actual experiments, to confirm the phase assemblage depending on the presence of limestone. The XRD patterns at 3, 7, and 28 days are shown in Figure 9. Unhydrated clinker phases and portlandite were commonly observed in all samples, so they were not presented in the XRD pattern. Instead, the peak range related to the chemical effects of limestone, 8–13°, was detailed.

In the system containing only clinker and gypsum (Figure 9a), ettringite and monosulfoaluminate were observed at all ages, while monocarboaluminate and hemicarboaluminate did not form. Additionally, as the age increased, changes in the peak intensity of ettringite and monosulfoaluminate were observed, with the peak of monosulfoaluminate becoming higher at a later age. However, peaks for monocarboaluminate and hemicarboaluminate were still not observed.

In the XRD analysis results (Figure 9b), where limestone was replaced by 2.5% up to 15% based on the plain, ettringite was observed at all ages, but the formation of monosulfoaluminate, as seen in Figure 9a, was not observed. On the other hand, the presence of limestone resulted in the formation of hemicarboaluminate and monocarboaluminate. At 3 days, the peak intensity of hemicarboaluminate was higher than that of monocarboaluminate, but this was reversed with increasing age, where the peak intensity of monocarboaluminate became higher.

#### 3.5.2. Thermodynamic Modeling

**Figure 10:**
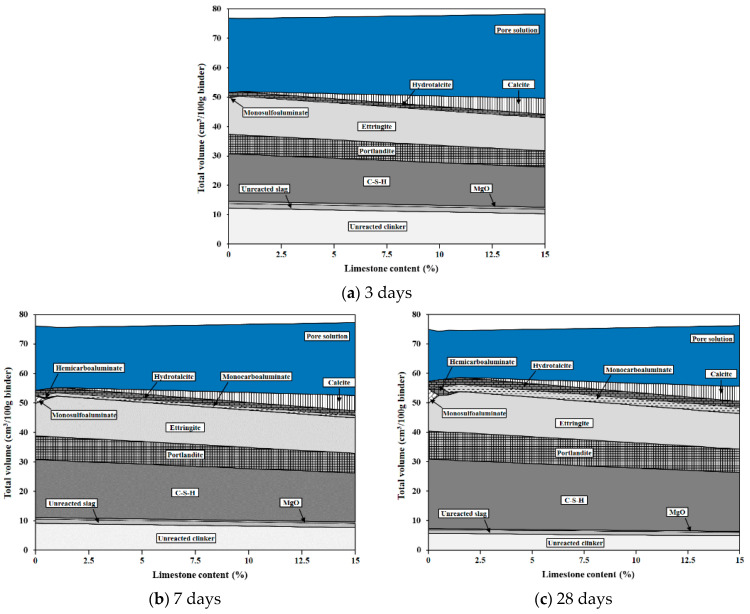
Phase assemblage of Portland cement by thermodynamic modeling for different amounts of limestone and reaction time.

Figure 10 shows the changes in the phase assemblage at 3, 7, and 28 days with increasing limestone content. The modeling results showed the formation of C–S–H and portlandite, which are typical hydrates of cement, as well as the formation of SO4-Aft/Afm, ettringite, monosulfoaluminate, CO_3_–Afm (hemicarboaluminate and monocarboaluminate), and the magnesium hydrate hydrotalcite [31]. C–S–H, portlandite, and ettringite were commonly present in all systems, but there were some differences in the phase assemblage depending on the age and the presence and amount of limestone.

In the system without limestone, a large amount of ettringite was formed at 3 days, and monosulfoaluminate was present in very small amounts. At 7 and 28 days, the amount of ettringite decreased while the amount of monosulfoaluminate increased. Since limestone was not present in the system, the formation of CO_3_–Afm phases did not occur. In systems with added limestone, the chemical effect of limestone was not observed up to 3 days, but CO_3_–Afm phases were observed at 7 and 28 days. The formation of hydrates showed the coexistence of hemicarboaluminate and monocarboaluminate up to 1% limestone content, but thereafter, only monocarboaluminate was present in the system. Meanwhile, when limestone was added, monosulfoaluminate was not present in the system, and the amount of ettringite increased.

## 4. Discussion

### 4.1. Flow

Because cement is ultimately used to manufacture concrete, its flow properties are one of the most important mechanical properties. In general, fluidity has a correlation with specific surface area. If the specific surface area is large, the area in contact with water increases, which increases water demand and leads to a decrease in fluidity and strength. Limestone is known to have a higher crushing rate than clinker [32]. Therefore, it can be assumed that within the co-crushed cement, limestone will be distributed in finer particles than the clinker. However, it is a positive result that fluidity was not significantly reduced even when limestone was used up to 15%. Prior research, including studies by Lee et al. (2003), highlighted limestone’s potential to enhance the particle size distribution of cement and, consequently, its flow properties [33]. Similarly, Sakai et al. (1997) identified that limestone powder could improve flow due to its superior particle morphology compared to cement [34]. The method used in this study had limitations in clearly explaining the effect of limestone particle size on fluidity, as shown in previous studies. This suggests the need for further research on the particle size distribution of limestone by manufacturing cement through separate grinding. It also indicates that future cement manufacturing should proceed with separate grinding to be advantageous in terms of quality control. Additionally, high-purity limestone was used in this study, meaning that the mineral composition of the limestone is mostly calcite and the amount of harmful components such as clay is small. In general, clay components have a high water absorption rate, which directly affects the flow characteristics [35]. Therefore, it is necessary to evaluate not only the physical properties of limestone but also the influence of mineralogical or chemical properties on fluidity [36].

### 4.2. Effect of Hydration Kinetics on Setting Time and Compressive Strength

The increase in limestone content clearly showed the promotion of hydration of C_3_S in the acceleration stage due to the filler effect, which is consistent with the results of previous studies [31]. The surface of finely ground limestone with clinker acts as a nucleus for the precipitation of hydrates [37,38]. Therefore, calcium ions in the pore solution were rapidly consumed to form C–S–H, and the degree of undersaturation of C_3_S increased, promoting hydration [14].

An intriguing result from this study is that the peak of C_3_A was not observed. Refs. [39,40] reported that when gypsum is present and the concentration of sulfate in the solution is high, sulfates are absorbed by the growing C–S–H. Additionally, when the concentration of sulfate ions in the solution decreases, sulfates can desorb from the C–S–H and form ettringite. Limestone promotes the hydration of early C_3_S due to the filler effect, leading to the precipitation of more C–S–H. Therefore, a faster depletion of solid gypsum may occur [41,42]. From this perspective, it is believed that the second peak of C_3_A in the heat flow might have occurred before or been integrated with the C_3_S peak.

Limestone reacts with C_3_A to form the CO_3_–AFm phase [6,31,43,44]. This formation reaction usually occurs after 24 h due to the low solubility of limestone and may not be clearly observed due to the low intensity and broad formation of peaks in the heat flow. However, in Figure 9b, limestone exhibited a chemical effect through its reaction with C_3_A by forming monocarboaluminate and hemicarboaluminate. This indicates that even though the second peak of C_3_A did not appear clearly, C_3_A had dissolved 24 h earlier and not all of it was consumed.

Heat of hydration results were found to correlate with setting time results. The initial setting of the cement occurs due to the precipitation of C–S–H at the end of the induction period and the beginning of the acceleration phase, with termination occurring within the acceleration phase [45,46]. The chemical effects of limestone do not affect the setting properties, as mentioned above. Therefore, the shortened setting time can be explained by the acceleration of hydration of C_3_S due to the filler effect of limestone [47].

It was expected that the acceleration of initial hydration due to the use of limestone would contribute to the improvement of the initial strength of cement mortar, but the strength at 1 day was approximately 2 MPa lower than that of the plain sample. As can be seen from the total heat release over 24 h, all samples showed lower heat release than the plain sample. Cement releases heat through hydration reactions, and higher heat release means more hydration products are formed. However, it is difficult to evaluate compressive strength based on heat release alone. Therefore, a discussion in terms of microstructure, such as pore structure, is necessary. On the other hand, after 3 days, the strength recovered to the same level as the plain sample or showed higher strength. The significant increase in strength development after 3 days is believed to be due to the chemical effect of limestone, which continuously participates in hydration reactions to form hemicarboaluminate and monocarboaluminate, contributing to strength development [48].

### 4.3. Phase Assemblages

The phase assemblage of hydrates formed through cement hydration can traditionally be confirmed through XRD and TGA. Recently, many researchers have been conducting various studies using thermodynamic modeling to predict hydrates more easily. Thermodynamic modeling is a fast and efficient analysis method that minimizes the time and effort spent during the curing period and preprocessing process because it does not require direct experimentation for analyzing cement hydrates. Additionally, it is possible to model the hydrates of cement with a predefined composition based on the results of XRF and XRD analysis, and variables such as temperature, pressure, fineness, curing environment, and the use of SCMs can be additionally applied. As a result, it is possible to predict the phase assemblage of cement hydrates, the mass and volume of hydrates over reaction time, and the changes in ions in the pore solution. This approach can be used to plan systematic experiments before conducting actual experiments and is useful for accurately evaluating the hydration reaction of cement by comparing and analyzing actual experimental results. Therefore, in this study, the chemical effect of limestone was evaluated by modeling the phase assemblage depending on the content of limestone, and it was compared and analyzed with actual XRD experimental results.

The filler effect and chemical effect of limestone both play important roles in the hydration reaction of cement. However, the use of limestone replaces reactive material; the focus should be on the chemical effect rather than the indirect effects of limestone. This chapter discusses the chemical effects of limestone based on modeling and XRD results.

The chemical effect of limestone is that C_3_A reacts with portlandite and limestone to form hemicarboaluminate and monocarboaluminate [43,49,50]. As seen in Figure 9a and Figure 10, these reactions do not occur in the absence of limestone [51]. Additionally, the reaction of limestone does not occur immediately after contact with water and cement but is activated after the consumption of gypsum in the system, leading to the precipitation of hydrates [43].

The formation of hydrates due to the chemical effect of limestone can be observed in the XRD results in Figure 9b at 3 days. Initially, the formation reaction of CO_3_–AFm phases was dominated by the formation of hemicarboaluminate, which can be explained by the difference in the solubility of limestone. In fact, the solubility of CaCO_3_ in limestone significantly decreases above pH 9, so the solubility of limestone in the highly alkaline conditions of the cement system is quite low, affecting the chemical effect of limestone [52,53]. Therefore, at an early age, due to insufficient carbonate in the pore solution, the precipitation of hemicarboaluminate occurs preferentially [31]. On the other hand, the modeling results in Figure 10a at 3 days did not show the formation of CO_3_–AFm phases under any conditions despite sufficient limestone content to form hemicarboaluminate, showing a different outcome compared to the experimentally analyzed XRD pattern.

The modeling results at 7 days confirmed the formation of CO_3_–AFm phases. However, due to the low solubility of limestone, hemicarboaluminate was present only up to 1% limestone content, and thereafter existed as monocarboaluminate. This also showed a discrepancy with the actual XRD results at 7 days. The XRD pattern in Figure 9b at 7 days confirmed that hemicarboaluminate was stably formed in all systems, and after 3 days, a partial transformation to monocarboaluminate was observed due to the additional dissolution of carbonates. This difference in the results between modeling and XRD analysis occurs because thermodynamic modeling calculates the stable phase assemblage based on the solution composition within the cement system [25,31,54]. The modeling results at 28 days showed a phase assemblage similar to that at 7 days, with an increase in the amount of precipitated hydrates as hydration continued. Moreover, the XRD pattern results also showed a more significant transformation to monocarboaluminate, evident from the differences in peak intensity.

The chemical effect of limestone is significant not only as a filler but also as a reactive material forming CO_3_–AFm phases and additionally plays a role in stabilizing ettringite within the cement system by preventing its conversion to monosulfoaluminate [10,55,56,57]. Since ettringite occupies more than twice the volume of monosulfoaluminate, maintaining ettringite stably in the cement system leads to improved porosity, which consequently affects compressive strength [44,58]. This impact of ettringite on volume is observable in the modeling results. Based on the modeling results at 28 days, the largest volume (58.56 cm³/100 g) was shown at 1.5% limestone content, and at this point, the volume of ettringite (13.81 cm³/100 g) was also the greatest. This is the result of ettringite stabilization without the phase transition to monosulfoaluminate after the addition of limestone. Moreover, from this point onwards, the system’s volume continuously decreased because, up to 1.5% limestone content, all limestone participates in the hydration reaction to form hydrates, but afterward, unreacted limestone remains in the system, causing a dilution effect of clinker.

Due to the considerably slow dissolution of limestone in the cement system, it is difficult to form a significant number of CO_3_–AFm phases at an early age. Additionally, the C_3_A in clinker that reacts with limestone is involved in the ettringite formation reaction through its reaction with gypsum, limiting the extent of limestone’s chemical effect. Therefore, if considering only increasing the chemical effect of limestone, replacing part of the clinker with SCMs containing alumina to enhance the reaction with limestone could be an effective method.

### 4.4. Determination of the Optimal Limestone Content in OPC

In Korea, the primary focus is on increasing the use of SCMs in OPC to reduce dependence on clinker. Therefore, research on the types and proportions of SCMs used is urgently needed. When comprehensively analyzing the results of this study, OPC with increased limestone content entails some dilution effect due to the reduced clinker content. However, considering limestone’s chemical effect and aspects of compressive strength development, it is believed that the optimal amount of limestone is appropriate to be within 10% under conditions blended with a small amount of GGBFS. Although all cement in this study was manufactured by co-grinding, limestone has a higher grinding efficiency compared to clinker, and the higher the fineness of limestone, the greater the reaction mechanisms. Therefore, if cement is manufactured through co-grinding, some of the effects of limestone within the cement could have been offset. As a solution to this issue, manufacturing cement by separately grinding could contribute to improving engineering properties such as the early strength of OPC containing limestone.

## 5. Conclusions

1. With increasing limestone content, the flow decreased slightly; however, there was no significant effect on workability. Furthermore, the flow was unaffected by limestone’s particle size distribution due to co-grinding. Additional research is required to explore how flow is influenced by limestone quality, including particle size distribution.
2. The filler effect of limestone accelerated the hydration of C_3_S after the induction period and shortened the time until the main peak. However, after 12–13 h, the dilution effect became more dominant, resulting in less heat emission than the plain cement. The clear C_3_A peak that occurs after gypsum consumption was not observed due to the decrease in clinker content.
3. As limestone content increased, setting times were shortened, with the initial limestone accelerating the hydration of C_3_S. The setting times were reduced significantly by 2 h.
4. After 1 day, the development of compressive strength was relatively low, despite the initial acceleration by the limestone’s filler effect. However, starting at 3 days, limestone chemically participated in the hydration reaction, forming CO_3_–AFm phases and stabilizing the ettringite, partially compensating for the strength reduction caused by the dilution effect. Notably, when 7.5% limestone was used, the test sample exhibited higher strength than the plain cement on all days after 3 days; at 28 days, even L10 developed higher strength than the plain cement.
5. By utilizing limestone and increasing limestone content, drying shrinkage was not negatively impacted. The greatest shrinkage occurred in the plain cement, and the shrinkage reduction effect increased with higher limestone content.
6. From the comparative analysis of limestone’s chemical effect through modeling and XRD patterns conducted in this study, both analytical methods confirmed that limestone participated in the chemical reaction through the formation of CO_3_–AFm phases and stabilization of ettringite. However, some differences in the types of phases formed depending on the age and limestone content were observed according to the analytical method.

## Figures and Tables

**Table 1:** Composition of raw materials used in cement manufacturing.

Sample	Materials (%)			
	Clinker	Gypsum	Limestone	GGBFS
Plain	85.5	4.5	5.0	5
L7.5	83.125	4.375	7.5	5
L10	80.75	4.25	10.0	5
L12.5	78.375	4.125	12.5	5
L15	76.0	4.0	15.0	5

Where L: limestone.

**Table 2:** Chemical compositions and density of raw materials.

Property		Material (%)			
		Clinker	Gypsum	Limestone	GGBFS
Density (g/cm^3^)		3.15	2.81	2.75	2.95
Chemical composition (%)	CaO	64.2	32.4	52.0	44.2
	SiO_2_	17.1	1.7	0.3	33.2
	Al_2_O_3_	4.0	0.7	0.1	13.2
	MgO	2.5	0.3	0.5	3.3
	Fe_2_O_3_	3.4	0.8	0.1	0.8
	K_2_O	1.3	0.1	0.02	0.6
	SO_3_	3.5	37.6	0.03	1.8
	L.O.I	2.1	24.4	40.7	0.92

**Table 3:** Physical properties and chemical compositions of manufactured cements.

Property			Material (%)			
		Plain	L7.5	L10	L12.5	L15
Physical	Density (g/cm^3^)	3.15	3.14	3.15	3.14	3.14
	Fineness (cm^2^/g)	3.693	3.825	3.823	3.940	4.001
	Mean (μm)	14.67	13.52	14.11	13.15	13.28
Chemical (%)	CaO	62.6	63.1	62.9	63.1	63.5
	SiO_2_	17.5	16.9	16.3	15.7	14.9
	Al_2_O_3_	4.7	4.6	4.4	4.3	4.2
	MgO	2.3	2.1	2.1	2.0	1.9
	Fe_2_O_3_	3.1	3.1	2.9	2.9	2.9
	K_2_O	1.2	1.2	1.0	1.0	1.0
	SO_3_	3.7	3.5	3.4	3.4	3.2
	L.O.I	3.4	4.1	5.5	6.4	7.3

**Table 4:** Relative compressive strength of Lab-OPC with different content of limestone.

Sample	Relative Compressive Strength, f/f_Plain_ (%)			
	1 Day	3 Days	7 Days	28 Days
Plain	100	100	100	100
L7.5	74.2	107.2	102.6	100.9
L10	67.7	93.3	96.1	101.9
L12.5	64.5	93.3	91.1	97.2
L15	62.9	87.5	80.9	88.7

## Data Availability

The data presented in this study are available on request from the corresponding author.
